# Supplementary material for: Fe3N Nanoparticle-Encapsulated N-Doped Carbon Nanotubes on Biomass-Derived Carbon Cloth as Self-Standing Electrocatalyst for Oxygen Reduction Reaction
Source: Nanomaterials (Basel). 2023 Aug 28;13(17):2439. doi: 10.3390/nano13172439 (PMC10489878; doi:10.3390/nano13172439)
Supplement: Supplementary file 1 [file nanomaterials-13-02439-s001.zip › nanomaterials-2550359-supplementary.pdf]

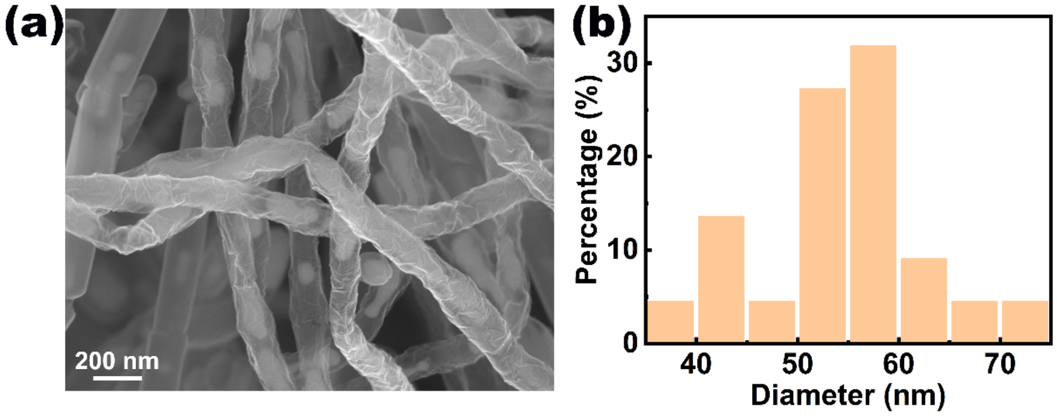

Figure S1. (a)STEM image of Fe3N@CNT/CC, (b)Histogram of the size distribution of nanoparticles.

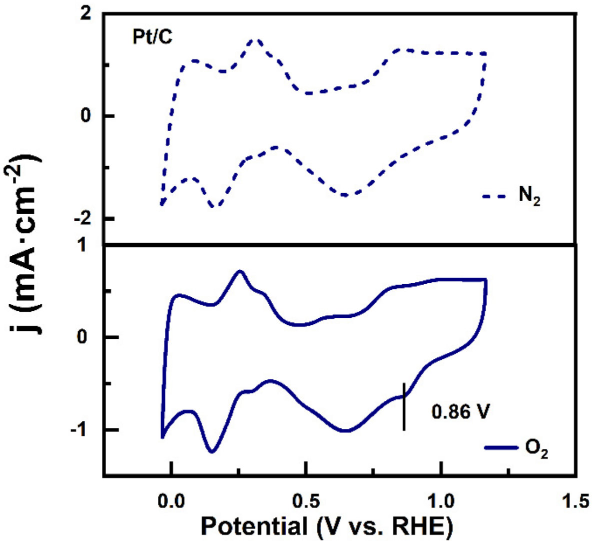

Figure S2. CV curves of the Pt/C in N_2 (dashed line) and O_2 (solid line) saturated 0.1 M KOH electrolyte with a scan rate of 10 $mV{\cdot}s^{-1}$.

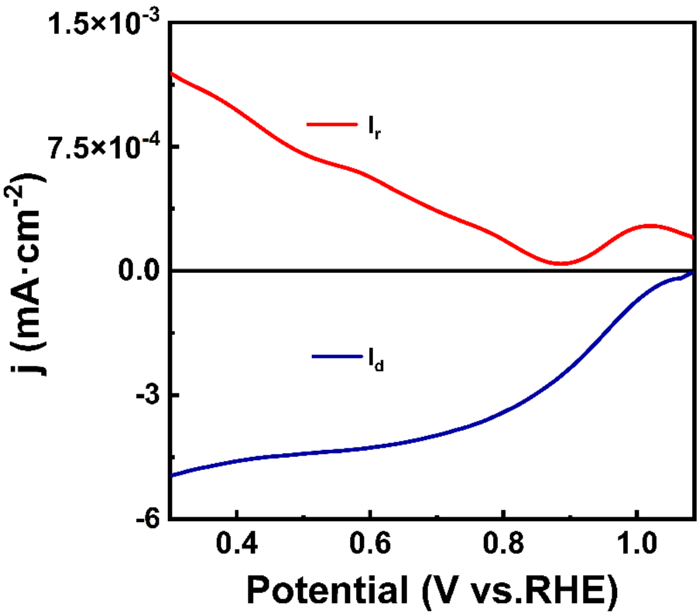

Figure S3. RRDE voltammograms of Fe_3N@CNT/CC at a rotation rate of 1600 rpm

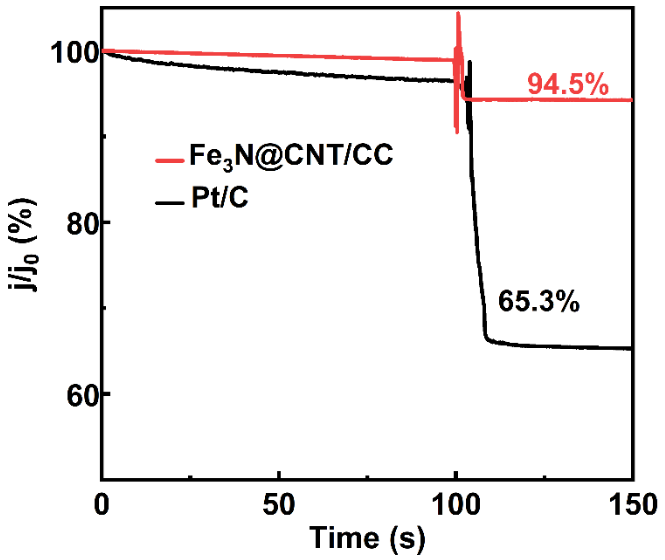

Figure S4. The chronoamperometric curves of Fe_3N@CNT/CC and Pt/C acquired by injecting 1.0 M methanol at 100 s

Table S1. Proportion of element content

Element	C	N	O	Fe
Weight fraction (%)	72.23	5.067	2.182	20.096
Molar fraction(%)	82.4	5.0	1.9	4.9

Table S2. Atomic percentage of C, N, O, Fe on the surface of Fe_3N@CNT/CC

Element	C	N	O	Fe
Molar fraction(%)	47.15	17.88	23.69	11.28
